# Acute Internal and External Loads in Change-of-Area and Fixed-Area 3v3v3+2 Small-Sided Games Across Three Pitch Sizes in Male Soccer Players

**DOI:** 10.3390/sports14100463

**Published:** 2026-10-19

**Authors:** Pakorn Chootsungnoen, Wirat Sonchan, Phornpot Chainok

**Affiliations:** Faculty of Sport Science, Burapha University, Chonburi 20131, Thailand; 66810004@go.buu.ac.th (P.C.); phornpot@go.buu.ac.th (P.C.)

**Keywords:** small-sided games, three-team rotation, transition play, pitch size, internal load, external load, global positioning system

## Abstract

Three-team rotation small-sided games (SSG) combine possession-based training with an embedded passive-recovery role, yet how the rule that governs attacking transitions interacts with pitch size in this format is unclear. This study used a repeated-measures design to compare the acute internal load, external load, and post-condition rating of perceived exertion (RPE) of two 3v3v3+2 SSG formats, change-of-area (COA) and fixed-area (FA), played on three pitch sizes (small, medium, and large; 75.3, 100, and 150 m^2^ per player). Each condition consisted of four 4 min bouts separated by 2 min of passive recovery. Twenty-two male soccer players (age 21.5 ± 1.85 years) took part. In COA, the team in possession redirected its attack toward a new target zone after a passing threshold was met; in FA, play remained in a single undivided zone. Heart rate (Polar H10), locomotor variables (10 Hz GPS, Catapult Vector S7), and RPE (modified Borg CR-10) were recorded. Format × pitch-size interactions were tested with repeated-measures ANOVA, and format differences within each pitch size were expressed as COA minus FA with 95% confidence intervals and Holm-adjusted *p*-values. The interaction was significant for HRmax, HRmean, time in the 70–79% and 90–100% HRmax zones, RPE, total distance, accelerations, and decelerations (all *p* < 0.05). On the small pitch, COA produced higher HRmax (185.5 ± 9.6 vs. 173.0 ± 7.1 bpm), RPE (7.9 ± 0.8 vs. 5.6 ± 0.8), total distance (1650.3 ± 280.8 vs. 1455.1 ± 299.0 m), accelerations, and decelerations (all Holm-adjusted *p* < 0.05, dz = 0.81–2.11), and on the medium pitch COA produced more high-speed running and decelerations. Higher heart-rate and total-distance values in FA on the medium and large pitches were suggestive but did not remain significant after adjustment. Within this protocol, COA was associated with a greater acute load on the small pitch, and the relative demand of the two formats varied with pitch size; these findings are provisional and apply to the studied male players and game structures.

## 1. Introduction

Soccer is an intermittent, high-intensity sport in which players must repeatedly and rapidly switch between attacking and defending roles under time pressure [1]. These transition phases, particularly the moments immediately after regaining or losing possession, can be among the most physically demanding passages of match play, with repeated high-speed running, sprinting, and abrupt deceleration, although these demands vary with possession duration, the location of ball loss or recovery, defensive organisation, and playing position [2,3,4,5]. In this study, transitions that follow a change in possession are distinguished from switching the point of attack during established possession, which is a different tactical situation. Small-sided games (SSG) are widely used to approximate these transition-specific demands within a controlled training environment because they combine technical, tactical, and physical loading in a single task [6,7,8,9].

Pitch size is one of the most influential constraints that coaches can manipulate when designing SSG because, together with player number, it determines the space available per player and, consequently, the physiological and locomotor demands imposed [10,11,12]. Enlarging the playing area has generally been associated with greater total distance and high-speed running, whereas smaller areas often increase the frequency of accelerations and decelerations, depending on the player-to-space ratio, the available running space, the game rules, and the measurement thresholds [10,13]. Total pitch area, pitch shape, and the effective area per active player are different quantities, and area per player is therefore used here as the primary spatial descriptor. Independently of pitch size, the tactical rule that triggers an attacking transition, for example, whether possession changes hands within a single fixed area or the attack must be redirected toward a new target zone, has been shown to alter the internal and external load elicited by otherwise similar SSG [14,15,16,17].

Beyond these two-team designs, coaches increasingly use continuous multi-team rotation formats, in which teams occupy attacking, defending, and resting roles in sequence, because they embed passive recovery in the drill and allow a larger squad to train together. A resting role reduces continuous playing exposure and can change the frequency of defensive transitions, the number of passing options, and the spatial organisation of the teams that are playing, and floating (neutral) players who support the team in possession may experience a different load from regular team players independently of the transition rule. A three-team rotation format (3v3v3+2) has been shown to be sensitive to the presence and initial positioning of an opposing team [18], suggesting that the internal structure of such games shapes their physical demands. However, because introducing a third, rotating team simultaneously changes player number, the numerical relationship between attackers and defenders, and the availability of passive within-bout rest, the effects of pitch size and transition rules observed in two-team formats cannot be assumed to generalise to a three-team rotation structure. In this study, COA required the team in possession to complete seven consecutive passes and then redirect the attack toward the opposite half, where the third team was waiting, whereas in FA play continued within a single area in which all three teams were active; the 3v3v3+2 format comprised three teams of three players and two neutral floating players who supported the team in possession.

Previous studies have examined pitch configuration, number of players, and the presence of an opposing team in transition games [14,16,18], but, to our knowledge, the combined effect of transition rule and pitch size within a continuous three-team rotation format has received little attention. Whether the relative load of COA and FA changes as area per player increases therefore remains unclear. The question matters because the physical cost of an SSG rule emerges from the combination of space, player numbers, and the rule itself rather than from any single constraint [10,13]. The two formats were compared across three pitch sizes using a 4 min work to 2 min passive-recovery structure (2:1 work-to-rest ratio), chosen for ecological validity in relation to training practice, not to simulate a complete microcycle [8]. The three pitch sizes provided 75.3, 100, and 150 m^2^ per player, spanning the small-to-large range of area-per-player values used in transition-focused SSG [19], in which larger areas per player have been reported to approximate the locomotor demands of official matches more closely [13].

Therefore, the purpose of the present study was to compare the acute internal load (heart-rate variables and post-condition RPE) and external load (total distance, high-speed running distance, sprint distance, peak speed, accelerations, and decelerations) responses to COA and FA formats within a 3v3v3+2 small-sided game across three pitch sizes (small, medium, large) in male soccer players. All outcome variables were specified in the research proposal before data collection; no single primary outcome was designated, so all outcomes were analysed with the same multiplicity correction. We hypothesised that pitch size would moderate the effect of game format on internal and external load, i.e., a significant format × pitch-size interaction. Because previous evidence does not allow a robust directional prediction at every pitch size, no a priori ordering of COA and FA was specified.

## 2. Materials and Methods

### 2.1. Participants

Twenty-two male soccer players (mean ± SD: age 21.5 ± 1.85 years, height 177.61 ± 6.09 cm, body mass 68.44 ± 7.61 kg, BMI 21.0 ± 1.79 kg·m^−2^, body fat 15.07 ± 3.41%, estimated V̇O_2_max 52.42 ± 1.48 mL·kg^−1^·min^−1^) were recruited for this study, classified as Tier 3 (highly trained/national-level) according to the participant classification framework of McKay et al. [20]. The players were professional players of a Thai League 3 club who trained five days per week and played one friendly or competitive match per week; the two-year competitive-experience requirement was an inclusion criterion, not the basis of the tier. Participants were recruited by purposive sampling, were free of orthopaedic or cardiovascular contraindications to exercise as confirmed by physician screening, had at least two years of competitive experience, and had not trained under hypoxic conditions in the six months preceding the study. Goalkeepers were excluded. The 22 players were organised into six fixed teams of three outfield players (18 players) and four neutral floating players. Players were ranked by estimated V̇O_2_max and assigned to teams so that no team differed statistically from another in aerobic fitness at baseline; the teams were formed after the Yo-Yo test, and their composition remained the same in all six sessions; technical–tactical skill and game knowledge were not assessed. Two 3v3v3+2 games were played simultaneously; each game comprised three teams of three players and two floating players (11 players per game; 2 × 11 = 22). All 22 players were included in the descriptive and inferential analyses, and a sensitivity analysis was performed on the 18 regular players. The sample size was calculated a priori with G*Power (version 3.1.9.4; Heinrich Heine University Düsseldorf, Düsseldorf, Germany) [21], as in previous repeated-measures small-sided-game studies [22]: for an effect size of 1.1, α = 0.05, and power = 0.95, the recommended total sample was 16 players. Twenty-two players were recruited because two simultaneous games of 11 players were required. The study was approved by the Human Research Ethics Committee of Burapha University (certificate no. IRB3-023/2569; project code G-HS105/2568; approved 10 March 2026) and was conducted in accordance with the Declaration of Helsinki. Written informed consent was obtained from all participants.

### 2.2. Experimental Design

A repeated-measures design was used in which all participants completed two 3v3v3+2 transition-based SSG formats: change-of-area (COA) and fixed-area (FA) at three pitch sizes (small, medium, large), yielding six experimental conditions in total, directly comparing the two game formats within each pitch size at a fixed 4 min work to 2 min passive-recovery ratio (2:1). The six conditions were completed in six sessions (one condition per session); the condition for each session was drawn by lot, so that all players performed the same condition in a given session (players had completed at least three familiarisation trials of the two formats beforehand), and a minimum 48 h recovery period separated consecutive sessions [23]. All sessions were conducted between 16:00 and 18:00 on an outdoor natural-grass soccer pitch, with ambient temperature between 31 and 34 °C in every session. Each session began with a standardised 15 min warm-up (5 min low-intensity jogging, 8 min dynamic stretching, 3 × 10 m maximal sprints), followed by four 4 min bouts of the assigned 3v3v3+2 format separated by 2 min of passive recovery, during which players stood and waited for the next bout. Multiple balls were kept pitch-side and immediately introduced whenever the ball left play to minimise interruptions; ball touches were unrestricted, and coaches provided consistent verbal encouragement throughout.

### 2.3. Game Formats

**Figure 1:**
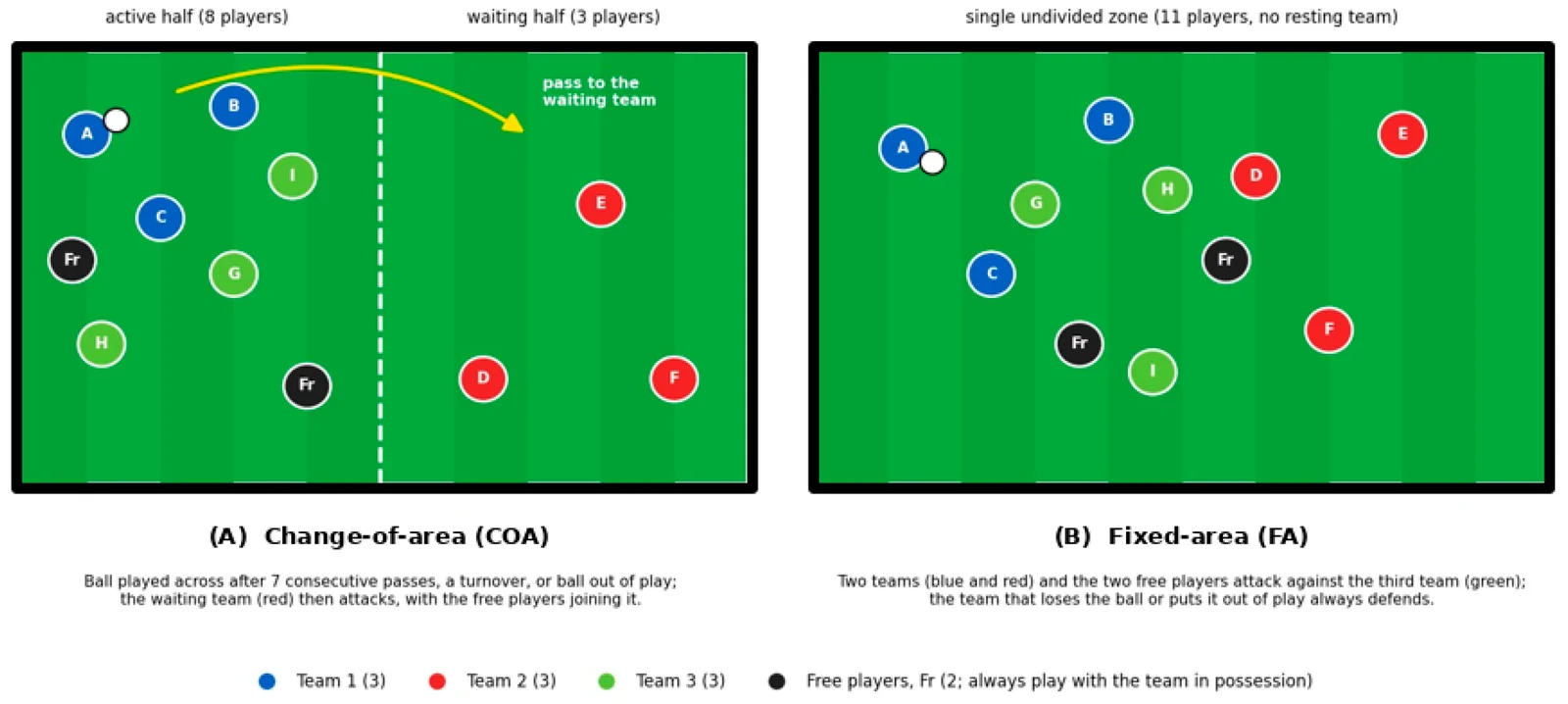
Schematic representation of the 3v3v3+2 formats (not to scale; blue, red, and green = the three teams of three players; Fr = free players): (**A**) change-of-area (COA), in which two teams of three and two free players play in one half of the pitch until seven consecutive passes are completed, after which the ball is played to the opposite half, where the third team of three is waiting; (**B**) fixed-area (FA), in which two teams and the two free players attack against the third team within a single undivided zone.

Two 3v3v3+2 games were played simultaneously in both formats, each comprising three teams of three players and two neutral floating players (11 players per game). For both formats, the small pitch measured 46 × 18 m (828 m^2^; 75.3 m^2^ per player), the medium pitch 55 × 20 m (1100 m^2^; 100 m^2^ per player), and the large pitch 66 × 25 m (1650 m^2^; 150 m^2^ per player), with area per player calculated for the 11 players in each game, consistent with area-per-player recommendations for transition-focused SSG [19]. In the change-of-area (COA) format, adapted from Asian-Clemente et al. [17], the pitch was divided into two halves. In one half, two teams of three players played 3v3, supported by two free players who always played with the team in possession (8 active players), while the third team of three players waited in the opposite half. The ball was played to the opposite half when the team in possession had completed seven consecutive passes, and also when the defending team won the ball or the ball went out of play. In every case, the waiting team received the ball and became the attacking team, with the free players joining it, and the defending team (after a turnover or a ball out of play, the team that had lost the ball or put it out) moved to the opposite half to contest the ball; when seven passes had been completed, the team that had made them stood and waited in the half where it had just played. In the fixed-area (FA) format, also adapted from [17], all 11 players played within a single, undivided zone (Figure 1B): two of the three teams (six players), together with the two free players (who always played with the team in possession), attacked and attempted to retain the ball for as long as possible against the third team (three players), which defended, so that no team rested. If the defending team won the ball while it remained in play, it was required to complete three consecutive passes before transitioning into an attacking role, and the team that had just lost the ball reverted to defending. If the ball went out of play, the team that had put it out likewise reverted to defending; in both formats, the team that lost the ball or put it out of play always became the defending team. Because attacking play in the FA format was never redirected toward a different physical target area, no directional “change in area” occurred, distinguishing it from the COA format.

### 2.4. Testing Procedures

Before each session, resting heart rate, blood pressure, and RPE were recorded. Body mass, height, and body fat percentage were assessed using a bioelectrical impedance body composition analyser (Omron BC-418, Omron Healthcare, Kyoto, Japan; reliability = 0.93) [24], and estimated maximal oxygen uptake was derived from the Yo-Yo Intermittent Recovery Test Level 1 using the equation of Karakoç et al. [25].

Internal load. Heart rate was recorded continuously using a chest-worn monitor (Polar H10, Polar Electro, Kempele, Finland; reliability = 0.93) [26]. Peak heart rate observed during the condition (HRmax), mean heart rate (HRmean), and the percentage of playing time spent in three heart-rate zones relative to individual reference HRmax (70–79%, 80–89%, 90–100%) were derived [27]. Zone times were expressed as a percentage of playing time (the four 4 min bouts, excluding the passive-recovery periods), as in previous SSG studies [27], and time below 70% HRmax was calculated as the remainder (100% minus the sum of the three zones). %HRmax and %HRmean were expressed relative to a common age-predicted maximum heart rate of about 199 bpm, calculated as 220 − age [28] (mean age of the sample 21.5 years, i.e., 220 − 21.5 ≈ 199 bpm); because age-predicted equations carry a considerable individual error [28,29], %HRmax values should be interpreted with caution. A single rating was obtained immediately after each condition (after the fourth bout), when players rated their perceived exertion using the modified Borg CR-10 scale (1 = very light, 10 = maximal effort) [30].

External load. External load was quantified using a 10 Hz global positioning system unit integrated with a tri-axial accelerometer (Catapult Vector S7, Catapult Sports, Melbourne, Australia), which has demonstrated acceptable validity and inter-unit reliability for distance and speed variables during team-sport locomotion [31,32,33,34]. Each player wore the same unit throughout the study in a manufacturer-fitted vest positioned between the scapulae, and devices were activated ≥15 min before each session to ensure satellite lock. Total distance covered (m), high-speed running distance (>19.8 km·h^−1^), sprint distance (>25.2 km·h^−1^), peak speed (m·s^−1^), and number of accelerations (>2.5 m·s^−2^) and number of decelerations (>2.5 m·s^−2^) [35]; data were processed with Catapult OpenField software (version 3.14.1, Catapult Sports, Melbourne, Australia); no additional minimum event duration was set by the authors beyond the software’s built-in event-detection criteria with user-defined velocity and acceleration thresholds as stated above (rather than the manufacturer’s default settings). Distance and event counts are totals for the whole condition (four bouts), and peak speed is the highest speed reached during the condition.

### 2.5. Statistical Analysis

Data are presented as mean ± SD. The effects of game format (COA, FA), pitch size (small, medium, large), and their interaction were examined for each outcome with a 2 × 3 repeated-measures ANOVA (Greenhouse–Geisser correction), with player as the repeated-measures unit; results were cross-checked with a linear mixed-effects model including a random intercept for player. Because the dataset contains one value per player and condition and no team or game identifiers, dependence among players within the same team or game could not be modelled. Format differences were then estimated within each pitch size with paired-samples *t*-tests and are reported as the mean difference (COA minus FA) with 95% confidence intervals and Cohen’s dz for paired data (trivial < 0.2, small 0.2–0.49, moderate 0.5–0.79, large ≥ 0.8) [36]. Normality of the paired differences was examined with the Shapiro–Wilk test for each of the 42 comparisons; it was rejected in 10 of them (mainly time in the 90–100% HRmax zone, sprint distance, and high-speed running on the small pitch), so Wilcoxon signed-rank tests were run for all comparisons as a check, and none of them changed the conclusion at the 0.05 level. To account for multiplicity across the 42 within-pitch comparisons (14 outcomes × 3 pitch sizes), *p*-values were adjusted with the Holm method; unadjusted *p*-values are also shown, but only Holm-adjusted results are described as statistically supported. The analysis was repeated in the 18 regular players (excluding the four floating players) as a sensitivity analysis. Statistical significance was set at *p* < 0.05. Analyses were performed in jamovi (version 2.6.17; The Jamovi Project, Sydney, Australia).

## 3. Results

Descriptive data for internal-load and external-load variables across the two game formats and three pitch sizes are presented in Table 1 and Table 2 (N = 22; no missing data). Format differences within each pitch size (COA minus FA) are presented in Table 3 (internal load) and Table 4 (external load). Heart-rate zone data are percentages of playing time; distances, event counts, and RPE refer to the whole condition.

The format × pitch-size interaction was significant for HRmax (F(2, 42) = 20.3), %HRmax (F = 20.5), HRmean (F = 10.0), %HRmean (F = 8.2), time in the 70–79% HRmax zone (F = 15.0), time in the 90–100% HRmax zone (F = 4.3), RPE (F = 14.0), total distance (F = 11.6), accelerations (F = 6.8), and decelerations (F = 9.1) (all *p* < 0.05), and was not significant for time in the 80–89% HRmax zone, high-speed running distance, sprint distance, or peak speed (all *p* > 0.25). The same conclusions were obtained when the analysis was restricted to the 18 regular players. After Holm adjustment across the 42 within-pitch comparisons, the differences that remained statistically supported were: on the small pitch, higher HRmax, %HRmax, RPE, total distance, accelerations, and decelerations, and less time in the 70–79% HRmax zone, in COA than in FA; and on the medium pitch, greater high-speed running distance and more decelerations in COA than in FA. The other differences described below were nominally significant (unadjusted *p* < 0.05) but did not remain significant after adjustment and are considered suggestive only.

On the small pitch, COA elicited a higher internal load than FA: HRmax (185.5 ± 9.6 vs. 173.0 ± 7.1 bpm; mean difference 12.5 bpm, *p* < 0.001, dz = 1.76), %HRmax (93.1 ± 4.8 vs. 86.8 ± 3.6%; *p* < 0.001, dz = 1.80), HRmean (161.3 ± 10.5 vs. 153.5 ± 8.3 bpm; *p* = 0.002, dz = 0.77; Holm-adjusted *p* = 0.056), and RPE (7.9 ± 0.8 vs. 5.6 ± 0.8; *p* < 0.001, dz = 2.11, the largest effect size observed in the study). The percentage of playing time in the 70–79% HRmax zone was greater in FA than in COA (36.7 ± 14.4% vs. 24.3 ± 9.1%; *p* < 0.001, dz = −1.12), whereas the percentage of playing time in the 90–100% HRmax zone was greater in COA (17.0 ± 23.3% vs. 2.5 ± 4.8%; *p* = 0.006, dz = 0.65), although this did not remain significant after adjustment (Holm-adjusted *p* = 0.189).

External load on the small pitch showed the same direction: total distance (1650.3 ± 280.8 vs. 1455.1 ± 299.0 m; *p* = 0.001, dz = 0.81), accelerations (20.0 ± 4.8 vs. 12.8 ± 6.1; *p* < 0.001, dz = 1.03), and decelerations (22.9 ± 8.2 vs. 13.2 ± 7.5; *p* < 0.001, dz = 1.18) were greater in COA than in FA and remained significant after Holm adjustment, whereas the difference in peak speed (5.6 ± 1.1 vs. 5.1 ± 0.6 m·s^−1^; *p* = 0.045, dz = 0.45) did not (Holm-adjusted *p* = 0.860). High-speed running and sprint distance were numerically higher in COA but did not differ significantly (*p* = 0.103 and *p* = 0.063, respectively).

On the medium pitch, HRmax (171.4 ± 12.6 vs. 177.7 ± 15.7 bpm; *p* = 0.037, dz = −0.47), %HRmax (*p* = 0.033, dz = −0.49), HRmean (*p* = 0.035, dz = −0.48), and %HRmean (*p* = 0.034, dz = −0.48) were nominally lower in COA than in FA, and the percentage of playing time in the 70–79% HRmax zone was greater in COA (31.5 ± 20.0% vs. 21.1 ± 11.5%; *p* = 0.041, dz = 0.46); none of these differences remained significant after Holm adjustment (adjusted *p* = 0.843 for all). For external load, high-speed running distance was more than three times greater in COA than in FA (23.3 ± 20.3 vs. 6.1 ± 14.8 m; *p* = 0.001, dz = 0.78; Holm-adjusted *p* = 0.048), and decelerations were greater in COA (26.9 ± 9.8 vs. 18.0 ± 11.1; *p* < 0.001, dz = 0.94; Holm-adjusted *p* = 0.009), whereas total distance did not differ significantly between formats (*p* = 0.081).

On the large pitch, HRmax (172.5 ± 10.9 vs. 180.0 ± 10.5 bpm; *p* = 0.018, dz = −0.54) and %HRmax (*p* = 0.026, dz = −0.51) were nominally lower in COA than in FA, FA produced a greater total distance (1888.0 ± 408.8 vs. 1729.6 ± 506.3 m; *p* = 0.016, dz = −0.56) and more playing time in the 80–89% HRmax zone (44.7 ± 20.0% vs. 33.8 ± 26.3%; *p* = 0.032, dz = −0.49), and COA showed more playing time in the 70–79% HRmax zone (34.1 ± 17.1% vs. 25.2 ± 13.7%; *p* = 0.016, dz = 0.56) and a higher peak speed (5.8 ± 0.9 vs. 5.4 ± 0.7 m·s^−1^; *p* = 0.033, dz = 0.49). None of the large-pitch differences remained significant after Holm adjustment (adjusted *p* = 0.482–0.843), and the total-distance difference was also not significant in the analysis restricted to the 18 regular players (*p* = 0.058). RPE did not differ significantly between formats on the medium (*p* = 0.308) or large (*p* = 0.598) pitch, in contrast to the small pitch.

## 4. Discussion

This study compared COA and FA formats within a three-team rotation small-sided game (3v3v3+2) across pitch sizes under a 4 min work to 2 min passive-recovery structure; to our knowledge, the combination of these factors has received little attention. The main finding was that pitch size moderated the difference between formats: the format × pitch-size interaction was significant for most heart-rate, RPE, total-distance, acceleration, and deceleration outcomes. On the small pitch, COA elicited a greater internal load, RPE, and external load than FA for several variables, and these differences remained significant after adjustment for multiplicity. On the medium and large pitches, the direction of several heart-rate and distance differences reversed in favour of FA, but these differences did not remain significant after adjustment and should be regarded as suggestive.

The COA and FA conditions represented two distinct transition-based game structures, rather than isolated manipulations of a single transition rule. They differed in the requirement to redirect the attack toward a new area and, as described in Section 2.3, also in spatial organisation, numerical relationships, floater placement, passing requirements, and recovery exposure. The COA rule, which requires a minimum number of consecutive passes before the attack is redirected toward the opposite end of the pitch, was intended to represent an attacking transition in which a team that has secured possession switches the point of attack before the opposing block can reorganise [1,2,4]; it shares some features with such situations but does not reproduce the time pressure, the distances between lines, the body orientation, or the goal-related consequences of match play. The FA rule, in contrast, kept play within a single undivided zone with all three teams active and a three-pass requirement after a change in possession [3,5]. Because the formats differ in several ways, the observed differences cannot be attributed to the change in area alone. The finding that the difference between formats depends on pitch size indicates that the physical cost of a given game structure should not be assumed constant across the range of pitch sizes used in practice [37].

The large small-pitch effects for HRmax, %HRmax, and RPE (dz = 1.76–2.11) may indicate that, when the 11 players of a 3v3v3+2 game share a pitch providing 75.3 m^2^ per player, the requirement to redirect the attack toward a distant target zone imposes a higher perceptual and cardiovascular demand than the fixed-zone structure of FA. Reducing the space available per player compresses interpersonal distances and is associated with more frequent, tightly coupled decision-making [37]. One possible explanation is that the requirement to redirect play adds cognitive and coordinative demands beyond cardiovascular strain [15,30,38]; however, no cognitive, tactical, or technical variables were measured, so this explanation remains a hypothesis. Similarly, the small pitch may have amplified the effect of the COA structure by reducing available space and increasing close-range interactions, but differences in player organisation, in the placement of floating players, and in the effective area available in each part of the pitch prevent attributing the response to pitch area alone; the area per player calculated from the whole pitch may not correspond to the effective area per player in each format. In COA, only one half of the pitch was active at a time, so the effective area per active player was smaller than the whole-pitch value, whereas in FA all 11 players shared the whole pitch.

On the medium and large pitches, FA showed numerically higher HRmax, HRmean, and (on the large pitch) total distance than COA, but these differences did not remain significant after adjustment for multiplicity, and the large-pitch total-distance difference was not significant when the floating players were excluded; the pattern is therefore suggestive. It is consistent with the view that the demands of an SSG rule depend jointly on space, player numbers, and rule structure [39], so that a format that is more demanding on a small pitch cannot be assumed to remain so as pitch size increases. Because low- and moderate-speed running, support actions, possession duration, and inter-player distance were not measured, we cannot conclude that FA promoted sustained off-ball running. The results suggest that FA may have imposed a greater sustained cardiovascular and volume-related demand at larger pitch sizes, whereas COA retained a more intermittent and mechanically discontinuous profile, with more decelerations and more high-speed running on the medium pitch and, nominally, a higher peak speed and more time in the 70–79% HRmax zone on the large pitch.

Decelerations were higher in COA than in FA on the small and medium pitches (dz = 0.94–1.18), and both differences remained significant after adjustment, whereas the large-pitch difference was not significant. GPS-derived deceleration counts describe the number of braking actions; they are not direct measures of eccentric force, neuromuscular fatigue, injury risk, or training adaptation, none of which was assessed. Horizontal deceleration is a mechanically demanding action in multidirectional sports [40,41], and formats that require players to decelerate, reorient, and re-accelerate toward a new target may therefore be of interest to coaches who wish to expose players to deceleration actions on small and medium pitches; whether this translates into adaptation or strain cannot be inferred from acute counts.

The between-format difference in RPE was significant only on the small pitch. In this protocol, a single post-condition RPE did not distinguish the formats on the medium and large pitches, suggesting that one global RPE measure may have limited sensitivity to the specific load differences produced by these formats; this does not show that RPE lacks sensitivity in general. RPE integrates cues beyond heart rate or distance, such as muscular effort, decision-making demand, and psychological engagement [30,38], but these were not measured here. Because RPE was collected once per condition, it may reflect the whole condition or the final bout, and a bout-by-bout analysis was not possible.

A further consideration is the work-to-rest structure applied in the present study. The 4 min work to 2 min passive-recovery ratio (2:1) used here provides comparatively little relative recovery between bouts, and recovery duration between SSG bouts has itself been shown to independently modulate both internal and external load, with shorter inter-bout recovery generally associated with a progressive rise in heart-rate-based internal load across bouts and a corresponding decline in high-intensity running output as a session progresses [42,43]. Because the present design fixed the work-to-rest ratio rather than manipulating it, the pitch-size-by-format interaction reported here should be interpreted as specific to a 2:1 recovery structure; whether the same interaction pattern would emerge under a more generous ratio such as 1:1, or would instead attenuate as within-session fatigue accumulates more slowly, remains an open question for future studies that manipulate recovery duration directly within this three-team rotation format. The scheduled 4:2 min work-to-rest ratio also differs from each player’s effective exposure: in the rotation format, active time depends on the player’s role, and the floating players in our data covered on average about 1009 m per condition compared with about 1864 m for regular players.

From a practical perspective, and provisionally, the results suggest the following. Where the aim is a high acute internal load together with more accelerations and decelerations on a small pitch (75.3 m^2^ per player), COA was associated with higher heart rate, RPE, acceleration, and deceleration values than FA. Where the aim is exposure to high-speed running and decelerations on a medium pitch (100 m^2^ per player), COA was associated with more of both. Where the aim is a higher total running volume on larger pitches, FA showed numerically higher values, although this was not statistically supported after adjustment. These are acute responses in this protocol and do not establish long-term training benefits. Sprint distances were very low in both formats (0.0–2.0 m per condition), so neither format should be regarded as a sprint-exposure drill on the basis of these data.

This study has several limitations. First, a single 4 min work to 2 min passive-recovery structure was used, and the results should not be generalised to other work-to-rest ratios or to active or heart-rate-controlled recovery [39]. Second, the four floating players were analysed together with the 18 regular players; their functional role and exposure differ (about 1009 m versus about 1864 m per condition), and the two formats also differed in floater placement, so the floater role may contribute to the format differences. A sensitivity analysis excluding the floaters gave the same interaction conclusions, but the large-pitch total-distance difference lost nominal significance. Third, groups were formed on estimated V̇O_2_max only; technical–tactical skill, motor capabilities, and game knowledge were not assessed, although players’ technical level, age, and biological maturity have been reported to shape their involvement and actions in small-sided games [44]. Fourth, the sample comprised male Thai League 3 players only, limiting generalisability to female, youth, or higher-level populations. Fifth, sessions were played outdoors on natural grass between 16:00 and 18:00 at 31–34 °C, and heat may have affected heart-rate and perceptual responses (humidity and wind were not recorded); learning and carry-over effects across sessions, despite a ≥48 h interval, cannot be excluded, even though players had completed at least three familiarisation trials; because the order of conditions was drawn by lot and was the same for all players, and session order is not part of the dataset, order effects could not be tested. Sixth, the data contain one value per player per condition, without team, game, or bout identifiers, so dependence among players in the same team or game and bout-to-bout variation could not be modelled, and confidence intervals may be too narrow. Seventh, technical and tactical variables, cognitive measures, and low- and moderate-speed running were not analysed [45,46,47]. Eighth, %HRmax was derived from an age-predicted maximum (220 − age) rather than a measured maximum, which carries a considerable individual error [28,29]. Finally, the number of comparisons was large, and only Holm-adjusted results are treated as statistically supported.

## 5. Conclusions

Within this three-team rotation small-sided game (3v3v3+2), the change-of-area (COA) and fixed-area (FA) formats differed in acute internal and external load, and the differences depended on pitch size (the format × pitch-size interaction was significant for most heart-rate, RPE, total-distance, acceleration, and deceleration outcomes). On the small pitch, COA was associated with higher HRmax, RPE, total distance, accelerations, and decelerations than FA; on the medium pitch, COA was associated with more high-speed running and decelerations; and the greater deceleration counts under COA were not significant on the large pitch. Higher heart-rate values in FA on the medium and large pitches and greater total distance in FA on the large pitch were suggestive only, because they did not remain significant after multiplicity adjustment, and the total-distance difference on the medium pitch was not significant. Because the formats differed in more than one game constraint and floating players had a different exposure, the findings should not be attributed to a single transition rule. They apply to the studied male players, game configurations, and session structure, and coaches may treat them as provisional guidance: COA may be useful when a higher acute load and more decelerations are desired on small and medium pitches, whereas the choice of format on larger pitches should depend on the training objective.

## Figures and Tables

**Table 1:** Internal load (mean ± SD) by game format and pitch size (N = 22).

Variable	Small COA	Small FA	Medium COA	Medium FA	Large COA	Large FA
HRmax (bpm)	185.5 ± 9.6 ***	173.0 ± 7.1 ***	171.4 ± 12.6 *	177.7 ± 15.7 *	172.5 ± 10.9 *	180.0 ± 10.5 *
%HRmax (%)	93.1 ± 4.8 ***	86.8 ± 3.6 ***	86.0 ± 6.2 *	89.2 ± 7.8 *	86.6 ± 5.4 *	90.2 ± 5.2 *
HRmean (bpm)	161.3 ± 10.5 **	153.5 ± 8.3 **	151.5 ± 15.6 *	157.7 ± 16.7 *	150.5 ± 14.6	156.5 ± 14.4
%HRmean (%)	80.6 ± 5.5 **	77.1 ± 4.1 **	76.0 ± 7.9 *	79.1 ± 8.2 *	75.5 ± 7.2	78.5 ± 7.2
Time 70–79% HRmax (% of playing time)	24.3 ± 9.1 ***	36.7 ± 14.4 ***	31.5 ± 20.0 *	21.1 ± 11.5 *	34.1 ± 17.1 *	25.2 ± 13.7 *
Time 80–89% HRmax (% of playing time)	42.6 ± 10.8	41.5 ± 19.0	30.8 ± 24.5	37.9 ± 23.4	33.8 ± 26.3 *	44.7 ± 20.0 *
Time 90–100% HRmax (% of playing time)	17.0 ± 23.3 **	2.5 ± 4.8 **	10.4 ± 20.0	16.4 ± 23.6	4.7 ± 9.1	8.6 ± 20.7
Time below 70% HRmax (% of playing time)	16.1 ± 11.1	19.2 ± 10.3	27.3 ± 23.6	24.6 ± 31.2	27.4 ± 24.1	21.5 ± 15.9
RPE (AU)	7.9 ± 0.8 ***	5.6 ± 0.8 ***	7.0 ± 1.3	6.7 ± 1.4	7.5 ± 1.5	7.4 ± 1.3

COA = change-of-area format; FA = fixed-area format; HR = heart rate. * *p* < 0.05, ** *p* < 0.01, *** *p* < 0.001 (unadjusted) for the paired-samples comparison between COA and FA within the corresponding pitch size (see Table 3 for adjusted *p*-values). Heart-rate zone values are % of playing time; time below 70% HRmax = 100% minus the three zones (descriptive).

**Table 2:** External load (mean ± SD) by game format and pitch size (N = 22).

Variable	Small COA	Small FA	Medium COA	Medium FA	Large COA	Large FA
Total distance (m)	1650.3 ± 280.8 **	1455.1 ± 299.0 **	1720.0 ± 349.4	1810.3 ± 465.3	1729.6 ± 506.3 *	1888.0 ± 408.8 *
High-speed running distance (m)	13.1 ± 20.1	4.9 ± 11.5	23.3 ± 20.3 **	6.1 ± 14.8 **	15.3 ± 11.8	9.2 ± 17.6
Sprint distance (m)	2.0 ± 4.8	0.0 ± 0.0	1.5 ± 4.9	0.5 ± 1.4	1.6 ± 4.1	0.5 ± 1.9
Peak speed (m·s^−1^)	5.6 ± 1.1 *	5.1 ± 0.6 *	5.7 ± 0.8	5.3 ± 0.9	5.8 ± 0.9 *	5.4 ± 0.7 *
Accelerations (n)	20.0 ± 4.8 ***	12.8 ± 6.1 ***	22.5 ± 10.8	19.2 ± 8.4	14.7 ± 6.1	17.4 ± 5.3
Decelerations (n)	22.9 ± 8.2 ***	13.2 ± 7.5 ***	26.9 ± 9.8 ***	18.0 ± 11.1 ***	16.8 ± 8.9	17.7 ± 8.7

COA = change-of-area format; FA = fixed-area format. * *p* < 0.05, ** *p* < 0.01, *** *p* < 0.001 (unadjusted) for the paired-samples comparison between COA and FA within the corresponding pitch size (see Table 4 for adjusted *p*-values).

**Table 3:** Pairwise comparisons (paired-samples *t*-test) between COA and FA formats within each pitch size for internal-load variables (N = 22).

Pitch Size	Variable	Mean Difference (COA − FA) [95% CI]	*p* (Unadjusted/Holm)	dz
**Large**	HRmax (bpm)	−7.5 [−13.7, −1.4]	0.018 */0.516	−0.54
	%HRmax (%)	−3.5 [−6.6, −0.5]	0.026 */0.697	−0.51
	HRmean (bpm)	−6.1 [−13.7, 1.6]	0.112/1.000	−0.35
	%HRmean (%)	−3.0 [−7.0, 0.9]	0.122/1.000	−0.34
	Time 70–79% HRmax (% of playing time)	8.9 [1.8, 15.9]	0.016 */0.482	0.56
	Time 80–89% HRmax (% of playing time)	−10.9 [−20.8, −1.0]	0.032 */0.843	−0.49
	Time 90–100% HRmax (% of playing time)	−3.9 [−14.5, 6.7]	0.451/1.000	−0.16
	RPE (AU)	0.2 [−0.5, 0.9]	0.598/1.000	0.11
**Medium**	HRmax (bpm)	−6.3 [−12.1, −0.4]	0.037 */0.843	−0.47
	%HRmax (%)	−3.2 [−6.1, −0.3]	0.033 */0.843	−0.49
	HRmean (bpm)	−6.2 [−11.9, −0.5]	0.035 */0.843	−0.48
	%HRmean (%)	−3.0 [−5.8, −0.2]	0.034 */0.843	−0.48
	Time 70–79% HRmax (% of playing time)	10.5 [0.4, 20.5]	0.041 */0.843	0.46
	Time 80–89% HRmax (% of playing time)	−7.1 [−18.3, 4.0]	0.197/1.000	−0.28
	Time 90–100% HRmax (% of playing time)	−6.0 [−17.0, 5.1]	0.274/1.000	−0.24
	RPE (AU)	0.3 [−0.3, 1.0]	0.308/1.000	0.22
**Small**	HRmax (bpm)	12.5 [9.4, 15.6]	<0.001 ***/<0.001	1.76
	%HRmax (%)	6.3 [4.8, 7.9]	<0.001 ***/<0.001	1.80
	HRmean (bpm)	7.7 [3.3, 12.2]	0.002 **/0.056	0.77
	%HRmean (%)	3.5 [1.2, 5.8]	0.004 **/0.128	0.69
	Time 70–79% HRmax (% of playing time)	−12.5 [−17.4, −7.5]	<0.001 ***/0.001	−1.12
	Time 80–89% HRmax (% of playing time)	1.1 [−10.2, 12.4]	0.843/1.000	0.04
	Time 90–100% HRmax (% of playing time)	14.5 [4.6, 24.4]	0.006 **/0.189	0.65
	RPE (AU)	2.3 [1.8, 2.8]	<0.001 ***/<0.001	2.11

dz = Cohen’s d for paired samples; * *p* < 0.05, ** *p* < 0.01, *** *p* < 0.001 (unadjusted). Differences are COA minus FA (percentage points for heart-rate zone data). The second *p*-value is Holm-adjusted across all 42 comparisons.

**Table 4:** Pairwise comparisons (paired-samples *t*-test) between COA and FA formats within each pitch size for external-load variables (N = 22).

Pitch Size	Variable	Mean Difference (COA − FA) [95% CI]	*p* (Unadjusted/Holm)	dz
**Large**	Total distance (m)	−158.5 [−284.3, −32.6]	0.016 */0.482	−0.56
	High-speed running distance (m)	6.1 [−1.6, 13.9]	0.116/1.000	0.35
	Sprint distance (m)	1.0 [−1.0, 3.1]	0.311/1.000	0.22
	Peak speed (m·s^−1^)	0.39 [0.03, 0.75]	0.033 */0.843	0.49
	Accelerations (n)	−2.7 [−5.6, 0.2]	0.069/1.000	−0.41
	Decelerations (n)	−1.0 [−5.2, 3.3]	0.647/1.000	−0.10
**Medium**	Total distance (m)	−90.3 [−192.5, 12.0]	0.081/1.000	−0.39
	High-speed running distance (m)	17.1 [7.4, 26.8]	0.001 **/0.048	0.78
	Sprint distance (m)	1.0 [−1.1, 3.0]	0.336/1.000	0.21
	Peak speed (m·s^−1^)	0.37 [−0.02, 0.75]	0.060/1.000	0.42
	Accelerations (n)	3.4 [−2.0, 8.7]	0.207/1.000	0.28
	Decelerations (n)	8.9 [4.7, 13.1]	<0.001 ***/0.009	0.94
**Small**	Total distance (m)	195.2 [88.7, 301.7]	0.001 **/0.036	0.81
	High-speed running distance (m)	8.2 [−1.8, 18.3]	0.103/1.000	0.36
	Sprint distance (m)	2.0 [−0.1, 4.1]	0.063/1.000	0.42
	Peak speed (m·s^−1^)	0.55 [0.01, 1.10]	0.045 */0.860	0.45
	Accelerations (n)	7.2 [4.1, 10.3]	<0.001 ***/0.003	1.03
	Decelerations (n)	9.7 [6.1, 13.4]	<0.001 ***/<0.001	1.18

dz = Cohen’s d for paired samples; * *p* < 0.05, ** *p* < 0.01, *** *p* < 0.001 (unadjusted). Differences are COA minus FA (percentage points for heart-rate zone data). The second *p*-value is Holm-adjusted across all 42 comparisons.

## Data Availability

The data that support the findings of this study are available from the corresponding author upon reasonable request.
